# Efficacy and safety of radiofrequency ablation for hypertrophic obstructive cardiomyopathy: A systematic review and meta‐analysis

**DOI:** 10.1002/clc.23341

**Published:** 2020-02-07

**Authors:** Haonan Yang, Yuan Yang, Yuzhou Xue, Suxin Luo

**Affiliations:** ^1^ Department of Cardiology The First Affiliated Hospital of Chongqing Medical University Chongqing China

**Keywords:** hypertrophic obstructive cardiomyopathy, meta‐analysis, radiofrequency ablation, septal reduction therapy

## Abstract

**Background:**

Although radiofrequency ablation is widely used in the treatment of arrhythmias, its role in septal reduction therapy of hypertrophic obstructive cardiomyopathy (HOCM) is unclear. This meta‐analysis aimed to assess the efficacy and safety of radiofrequency septal ablation for HOCM.

**Hypothesis:**

Radiofrequency septal ablation is effective and safe for relieving obstruction and improving exercise capacity in patients with HOCM.

**Methods:**

A systematic review of eligible studies that reported outcomes of patients with HOCM who underwent radiofrequency septal ablation was performed using PubMed, Embase, Cochrane, ProQuest, Scopus, ScienceDirect, and Web of Science database. Pooled estimates were calculated using random‐effects meta‐analysis. Methodological quality was assessed using the Newcastle‐Ottawa scale. Publication bias and sensitivity analyses were also performed.

**Results:**

Eight studies with 91 patients (mean follow‐up 11.6 months) were included. The left ventricular outflow tract (LVOT) gradient at rest decreased significantly after radiofrequency septal ablation (pooled reduction: −58.8 mmHg; 95% confidence interval [CI] −64.3 to −53.5). A reduction was also found in the provoked LVOT gradient with a pooled reduction of −97.6 mmHg (95% CI: −124.4 to −87.1). An improvement of the New York Heart Association classification (mean: −1.4; 95% CI: −1.6 to −1.2) was found during follow‐up. The change in septal thickness was minimal and not statistically significant. Two procedure‐related deaths were documented, and complete heart block occurred in eight patients.

**Conclusions:**

Radiofrequency septal ablation is effective and safe for relieving LVOT obstruction and improving exercise capacity in patients with HOCM.

## 1 INTRODUCTION

Hypertrophic obstructive cardiomyopathy (HOCM) could lead to symptoms of dyspnea, chest pain, and syncope due to left ventricular outflow tract (LVOT) obstruction. Population‐based studies estimated that the prevalence of HOCM was 0.02% to 0.23% in adults.^1^ The morbidity and mortality were higher in a population with LVOT obstruction than in that without.^2^, ^3^, ^4^

An increased LVOT gradient, which is defined as a peak gradient ≥30 mmHg, was present in 20% to 30% and 70% of hypertrophic cardiomyopathy patients at rest and with exercise provocation, respectively. The key causes of an increased LVOT gradient were septal hypertrophy and systolic anterior motion (SAM) of the mitral valve.^1^, ^5^ For decades, myectomy has been the golden standard therapy for symptomatic drug‐resistant HOCM because of its excellent outcome.^6^ Moreover, alcohol septal ablation (ASA) has been an appealing alternative approach in the treatment of HOCM because it effectively reduced symptoms with less trauma and shorter hospital stay than myectomy. Nevertheless, the use of ASA is limited by coronary anatomy, which means that 5% to15% of patients are unsuitable to undergo ASA.^7^, ^8^

Radiofrequency catheter ablation has been a well‐accepted treatment of cardiac arrhythmia for many decades. This technique has been also used to relieve LVOT obstruction without restriction of the coronary anatomy.^9^ However, the efficacy and safety of radiofrequency septal ablation have not been clear, as only small studies on this topic were conducted. Therefore, in the current study, we aimed to perform a systematic review and meta‐analysis of the short‐term efficacy and safety of radiofrequency ablation for HOCM.

## 2 METHODS

### 2.1 Search strategy

This systematic review and meta‐analysis was performed in accordance with the Preferred Reporting Items for Systematic Reviews and Meta‐Analyses guidelines and Meta‐analysis of Observational Studies in Epidemiology statement.^10^, ^11^ We searched PubMed, Embase, Cochrane, ProQuest, Scopus, ScienceDirect, and Web of Science database from January 1979 to July 2019 using the medical subject heading (MeSH) terms and all synonyms for hypertrophic cardiomyopathy in combination with the MeSH term and all synonyms for radiofrequency ablation (Supporting Information). In order to achieve maximum sensitivity and identify all relevant studies, we applied no language restrictions, and conference abstracts were considered. References to all identified publications were entered into reference‐managing software (Endnote, version X9; Clarivate Analytics, Philadelphia, Pennsylvania).

### 2.2 Study election and data extraction

Clinical studies that reported patients with typical HOCM who underwent radiofrequency ablation for HOCM were screened for inclusion. Typical HOCM was defined as LVOT gradient ≥50 mmHg (at rest or after provocation) and symptomatic despite adequate medical therapy. Exclusion criteria were as follows: studies that included myectomy and ASA, studies without a definite diagnosis, studies without a measurement of the LVOT gradient before or after radiofrequency ablation, reviews, case reports, editorials, and commentary articles. Two independent reviewers reviewed the study titles and abstracts according to the inclusion and exclusion criteria. Studies that satisfied the inclusion criterion were retrieved for full‐text assessment. Because of a lack of randomized controlled trial, we included only observational studies in the analysis.

The outcomes were as follows: change in the resting and provoked LVOT gradient, septal thickness, and New York Heart Association (NYHA) functional classification between baseline and post‐procedure. We extracted the following data from each selected study: the total number of participants, age, sex, duration of follow‐up, ablation site, and major complications. We also collected baseline and post‐procedure resting LVOT gradient values, provoked LVOT gradient value, NYHA class, and septal thickness.

### 2.3 Assessment of study quality

The Newcastle‐Ottawa scale (NOS)^12^ was used to assess the quality of each study, and included the selection of cases, comparability of cases and controls, and ascertainment of exposure to risks. Furthermore, a sensitivity analysis was considered as an essential part of the quality assessment.^10^ The quality of each study was assessed independently by two reviewers. Disagreements between the reviewers were resolved through discussion by the research group until a consensus was reached.

### 2.4 Statistical analysis

The LVOT gradient, septal thickness, and NYHA class were analyzed as continuous variables (mean ± SD). Pooled estimates of the mean difference in resting and provocation LVOT gradients were calculated by using a random‐effect model to describe the effect of radiofrequency septal ablation. We also calculated the pooled estimate of the mean difference in improvement of the septal thickness and NYHA class with a random‐effect model.

Heterogeneity of the included studies was examined by using the *Cochran Q* test, with *P* < .1 regarded as being statistically significant between‐study heterogeneity. The *I* ^2^ test was also utilized to assess the magnitude of heterogeneity between the studies; values >50% were regarded as indicating a moderate to high level heterogeneity.^11^ Further, sensitivity analyses were conducted by excluding one study sequentially, and the impact of removing each of the studies was evaluated on the basis of the pooled results and between‐study heterogeneity. The results were determined as credible when the pooled results and between‐study heterogeneity were not substantially different in the sensitivity analyses.

Additionally, publication bias was examined by constructing a funnel plot, and Begg's test and Egger's test were used to assess the funnel plot asymmetry. Statistical analysis of this meta‐analysis was conducted in Stata 15.0 (StataCorp, College Station, Texas).

## 3 RESULTS

### 3.1 Systematic review and study characteristics

**Figure 1:**
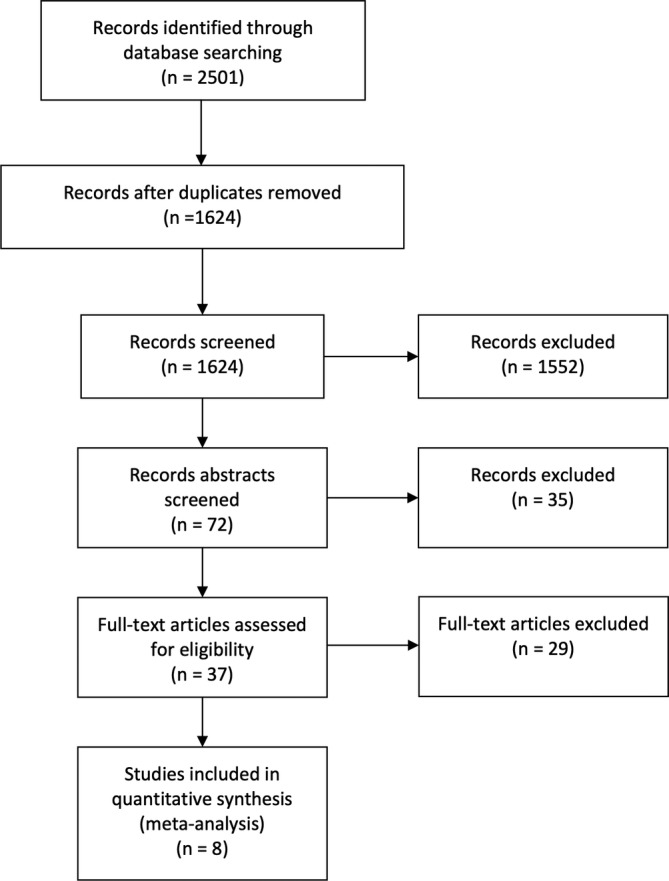
Flow diagram of study selection process

The database search identified 2501 records. After removing duplicates, 1624 studies remained. Eight studies^13^, ^14^, ^15^, ^16^, ^17^, ^18^, ^19^, ^20^ published between 2005 and 2019 (including a conference abstract) were included in this analysis according to the inclusion and exclusion criteria (Figure 1). Ninety‐one patients underwent radiofrequency septal ablation for HOCM with a mean follow‐up of 11.6 months (range, 1.3‐48 months). Patients' mean age was 44.2 years, and this result includes two studies that treated children (Table 1). All studies measured the LVOT gradient at rest (mean 77.8 mmHg). Each patient enrolled in these studies was diagnosed as having typical HOCM with an LVOT gradient ≥50 mmHg at rest or after provocation (Table 2). Four studies measured the provoked LVOT gradient before and after the procedure. Five studies reported septal thickness measured by echocardiography before the procedure, and four of them reported septal thickness post‐procedure. The NYHA class was reported in six studies that assessed the resolution of symptoms after radiofrequency septal ablation. All studies except one used a left‐sided approach (Table 3). Unlike other studies, Liu et al^20^ conducted percutaneous intramyocardial septal radiofrequency ablation in which the radiofrequency needle was inserted through the left ventricular apex.

**Table 1:** Characteristics of included studies

Author	Year	N	Mean age	Male/female	Follow‐up	Previous SM	Previous ASA	Reason for excluding SM/ASA	Complications
Emmel et al^13^	2005	3	11	N	1.5 mo	None	None	Technically unfeasible or risky	One VF
Sreeram et al^14^	2011	32	11.1	19/13	48 mo	1	None	N	One paradoxical increase in LVOTG (death); 2 PPM; 2 VF
Lawrenz et al^15^	2011	19	60.7	N	6 mo	None	8	Patient choice	One tamponade; 4 PPM
Shelke et al^16^	2014	7	43.7	5/2	12 mo	None	None	Unsuitable for ASA or surgical risk	One pulmonary edema
Cooper et al^17^	2015	5	57.6	1/4	6 mo	None	5	Surgical risk	One retroperitoneal hemorrhage (death); One pulmonary edema (LVOTG increase)
Crossen et al^18^	2016	11	62	4/7	12 mo	1	3	N	One tamponade; one pulmonary congestion; 2 PPM
Beaser et al^19^	2018	5	61	2/3	1.3 mo	None	None	Surgical risk	N
Liu et al^20^	2019	9	46.1	8/1	6 mo	None	None	N	No major complication

Abbreviations: ASA, alcohol septal ablation; N, not documented; PPM, permanent pacemaker; SM, surgical myectomy; VF, ventricular fibrillation.

**Table 2:** Baseline and post‐ablation clinical outcomes

Author	Baseline resting LVOTG (mmHg)	Baseline provoked LVOTG (mmHg)	Baseline septal thickness (mm)	Baseline NYHA class	Post‐ablation resting LVOTG (mmHg)	Post‐ablation provoked LVOTG (mmHg)	Post‐ablation septal thickness (mm)	Post‐ablation NYHA class
Emmel et al^13^	86.7 ± 5.8	N	N	N	26.7 ± 2.9	N	N	N
Sreeram et al^14^	96.9 ± 27	N	N	N	32.7 ± 27.1	N	N	N
Lawrenz et al^15^	77.7 ± 30	157.5 ± 37	22.6 ± 3.7	3.0	26.5 ± 22	64.2 ± 44	21.4 ± 3.4	1.6 ± 0.7
Shelke et al^16^	81.0 ± 14.8	N	N	3.0	42.8 ± 26.1	N	N	1.6 ± 0.8
Cooper et al^17^	64.3 ± 50.6	93.5 ± 30.9	18.3 ± 1.9	3.0	12.3 ± 2.5	23.3 ± 8.3	16.8 ± 2.5	1.8 ± 0.5
Crossen et al^18^	66.7 ± 39.5	136.2 ± 60.9	21.0	3.0	10.0 ± 5.7	20.0 ± 16.7	20.0	1.8 ± 0.8
Beaser et al^19^	65.6 ± 37.8	N	19.8 ± 4.5	3.0	10.4 ± 10.6	N	N	1.4 ± 0.9
Liu et al^20^	83.3 ± 32.4	147.8 ± 58	21.5 ± 2.6	2.8 ± 0.4	11.8 ± 5.7	25.5 ± 11.4	12.9 ± 1.9	1.3 ± 0.5

Abbreviations: LVOTG, left ventricular outflow tract gradient; N, not documented.

**Table 3:** Summary of procedural details

Author	Access	Catheter	Mapping system	Ablation site	Mean no. of lesions	Power (W)	Temperature (°C)	Duration of ablation lesions (s)	Total ablation duration (min)	Fluoroscopy time (min)	Procedure time (min)
Emmel et al^13^	Retrograde trans‐aortic access	Cooled‐tip ablation catheter	LocaLisa	LV	37.3	N	N	60	N	20.3	N
Sreeram et al^14^	Retrograde trans‐aortic access	4‐mm cooled‐tip ablation catheter in 30/32 8‐mm ablation electrode catheter in 2/32	LocaLisa in 30/32 CARTO in 2/32	LV	27	60	40 to 50	60 to 120	N	24	N
Lawrenz et al^15^	Retrograde trans‐aortic access	4‐mm irrigated‐tip ablation catheter	CARTO	LV in 9/19 RV in 10/19	31.2	54.7	N	90	N	N	139 ± 47
Shelke et al^16^	Retrograde trans‐aortic access	3.5‐mm irrigated‐tip ablation catheter	CARTO	LV	22.2	30 to 40	N	60 to 120	N	N	N
Cooper et al^17^	Retrograde trans‐aortic access	Navistar and THERMOCOOL catheter	CARTO	LV	N	50 to 60	60	N	33.6	N	N
Crossen et al^18^	Retrograde trans‐aortic access	4‐mm open‐irrigated ablation catheter	NavX	LV	43 ± 12	50	45	120	N	37.3 ± 11	142 ± 33
Beaser et al^19^	Atrial trans‐septal access	Irrigated‐tip ablation catheter	CARTO	LV	N	N	N	N	27.6	N	N
Liu et al^20^	Percutaneous intramyocardial approach	Cooled‐tip ablation needle	None	Intraseptal	N	60 to 100	N	N	61.3 ± 18.8	N	N

Abbreviations: ASA, alcohol septal ablation; N, not documented; SM, surgical myectomy.

### 3.2 Quality assessment of included studies

All studies were assessed by NOS in which two studies^13^, ^19^ received seven scores, four studies^14^, ^15^, ^16^, ^20^ received eight scores and two studies^17^, ^18^ received nine scores. It represented moderate to high methodological quality.

### 3.3 Clinical efficacy and safety of radiofrequency ablation

**Figure 2:**
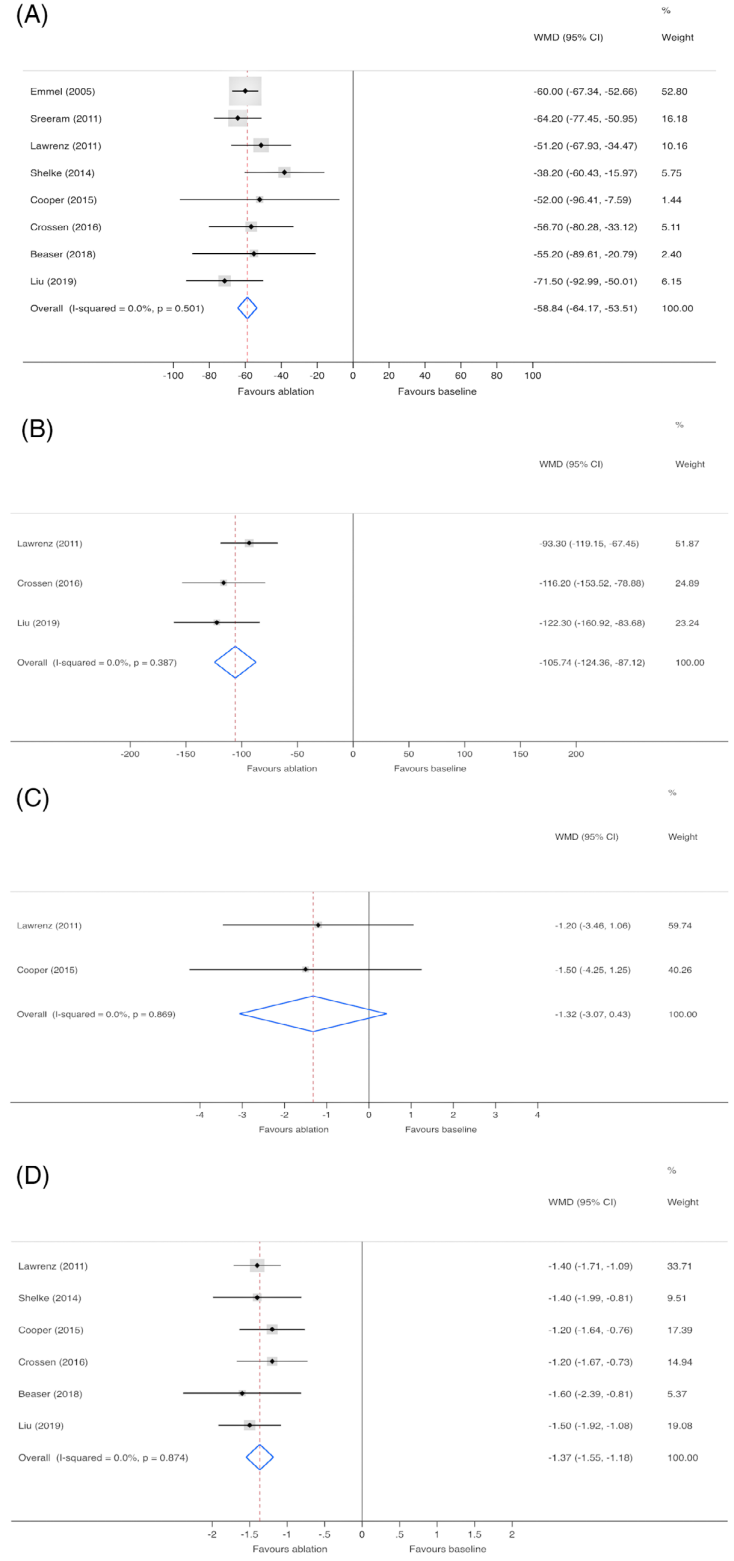
Forest plots of the resting LVOT gradient, A, the provoked LVOT gradient, B, septal thickness, C, the NYHA class, D. For each estimate, the gray shaded area is the weight of the estimate in proportion to the overall effect. CI, confidence interval; LVOT, left ventricular outflow tract; NYHA, New York Heart Association; WMD, weighted mean difference

Patients' mean resting LVOT gradient was reported to be significantly reduced in all eight studies, whereas the average decrease in this parameter differed from 38.2 to 71.5 mmHg in different studies. In a pooled analysis of all eight studies (Figure 2), mean reduction of the resting LVOT gradient was 58.8 mmHg (*P* < .01), with no statistically significant between‐study heterogeneity. Furthermore, every study reported a mean resting LVOT gradient ≥30 mmHg after radiofrequency septal ablation. Pooled analysis of four studies that reported the provoked LVOT gradient of participants showed a 97.6‐mmHg reduction (*P* < .01) after the procedure with moderate between‐study heterogeneity. Sensitivity analysis showed a reduction in heterogeneity when the study conducted by Cooper et al^17^ was excluded. Thus, another pooled analysis containing three studies (Figure 2) was conducted and showed a reduction in the provoked LVOT gradient of 105.7 mmHg (*P* < .01) after radiofrequency ablation.

There was minimal change in septal thickness after patients underwent radiofrequency septal ablation. Pooled analysis of septal thickness (Figure 2) showed only a mean reduction in septal thickness of 1.3 mm (*P* = .14), including two studies that reported reductions of 1.2 and 1.5 mm, respectively. All studies documented symptom relief or improvement of the NYHA classification during the follow‐up. Pooled analysis of the six studies (Figure 2), which estimated the NYHA class of patients, showed that the mean improvement in the NYHA class was 1.4 after the procedure.

### 3.4 Complications associated with radiofrequency ablation

Major complications were documented in six clinical studies, whereas the others did not document complications or only documented a mild complication. Two procedure‐related deaths due to retroperitoneal hemorrhage and paradoxical increase in the LVOT gradient caused by tissue edema after ablation were documented among 91 participants. Complete heart block occurred in eight (8.8%) patients with permanent pacemaker dependency, and three patients developed ventricular fibrillation requiring cardioversion.

### 3.5 Sensitivity analysis and publication bias

**Figure 3:**
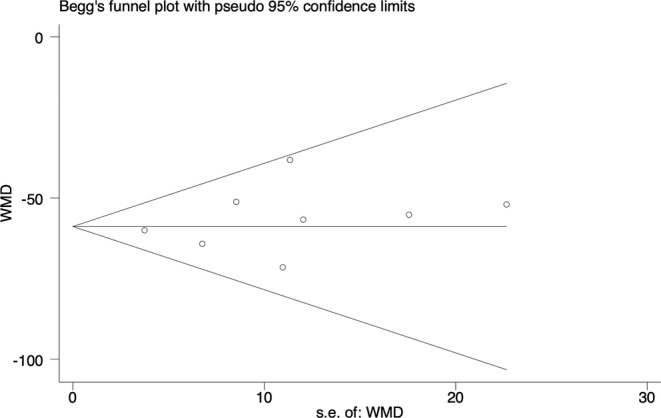
Begg's test funnel plot for the resting LVOT gradient. LVOT, left ventricular outflow tract

As most studies did not measure septal thickness before or after the procedure, pooled analysis of septal thickness included only three studies, which led to severe heterogeneity. Sensitivity analyses found that the corresponding pooled results and heterogeneity were not significantly different in all analyses, except for the pooled analysis of the change in septal thickness after ablation. When Liu et al's study^20^ was excluded from the analysis, the pooled mean differences of septal thickness and heterogeneity were reduced significantly (*I* ^2^ test: 93‐0%, *X* ^2^ test: *P* < .01 to *P* = .87). Thus, the final pooled analysis of change in septal thickness did not include the study conducted by Liu et al.^20^ No publication bias was evident for any of the pooled analyses. An example of Begg's test funnel plot for resting LVOT gradient is shown in Figure 3.

## 4 DISCUSSION

Septal reduction therapy is essential when optimal medication therapy cannot relieve patients' symptoms. Although the benefits of radiofrequency ablation have been widely accepted in the treatment of cardiac arrhythmias, such as ventricular tachycardia and atrial fibrillation, it is still a novel technique in patients with HOCM. Lawrenz et al^21^ first reported the use of radiofrequency ablation in a 45‐year‐old patient with severe HOCM. They also used a right‐sided approach in another studies that did not indicate any difference between the right‐sided and left‐sided approaches.^15^ Several case reports of radiofrequency septal ablation have been documented since then, with a promising result in reducing the LVOT gradient.^22^, ^23^, ^24^ Eight studies with 91 participants were included in the current meta‐analysis. Since all the included studies were assessed by scales and sensitivity analyses during the quality assessment process, we concluded that the results on the basis of current evidence were relatively convincing.

Radiofrequency septal ablation was effective in reducing resting and provoked LVOT gradients of patients with HOCM compared with baseline values, and reduction of the provoked LVOT gradient might be greater than the resting LVOT gradient. Furthermore, reduction of the LVOT gradient resulted in significant improvement of patients' symptoms and exercise capacity. The heterogeneity and publication bias of the pooled analyses were not significant after conducting the sensitivity analyses. Compared with ASA and myectomy, which could lead to reductions of 71% and 77%, respectively, in the LVOT gradient after the procedures,^25^ radiofrequency ablation resulted in a pooled reduction of 75.6% in the LVOT gradient, which was reasonable. Although the change of septal thickness could be up to 5 to 6 mm in ASA and more than 8 mm in myectomy,^26^, ^27^, ^28^ there was minimal change in septal thickness after radiofrequency ablation, with only two studies that reported a reduction <2 mm. The SAM of the mitral valve contacting the hypertrophied septum in mid‐systole was the key mechanism of LVOT obstruction.^29^ Moreover, this SAM‐septal feedback mechanism would produce a >30 mmHg pressure gradient at rest or after provocation. Thus, in theory, septal reduction, which stopped the SAM‐septal feedback mechanism, could reduce the LVOT gradients. With the electroanatomical mapping system, the advantage of radiofrequency septal ablation was accuracy in targeting the SAM‐septal contact area, independent of the coronary anatomy. Studies^15^, ^17^ proved that a small amount of damage accurately delivered through radiofrequency ablation did interrupt the SAM‐septal feedback mechanism and reduced the LVOT gradient as effectively as ASA, with a minimal change in septal thickness.

Although it has been more than 20 years since the first introduction of ASA, there is still concern about its safety, especially the complete heart block risk. A meta‐analysis^25^ reported that the periprocedural mortalities were 1.3% for ASA and 2.5% for myectomy. Although lower mortality was observed after ASA, the proportion of permanent pacemaker dependency after ASA was 10% compared with 4.4% after myectomy.^25^ In this review, we found that the incidence of permanent pacemaker implantation was 8.8%, which was not higher than that of ASA. However, clinical studies of radiofrequency ablation conducted in recent years reported a lower incidence of pacemaker dependency because of use of the electroanatomical mapping system.^16^, ^17^, ^18^ The His conduction tissue, left bundle, and left anterior and posterior fascicles were directly mapped during radiofrequency ablation, which helped to preserve atrioventricular conduction and was a potential benefit of radiofrequency ablation. Nevertheless, more clinical studies are expected to support that radiofrequency septal ablation is beneficial. Periprocedural mortality (2.2%) in this meta‐analysis was similar when compared with that of ASA and myectomy. Two cases of paradoxical increase in the LVOT gradient after the procedure were reported among 91 patients, and one of those cases resulted in death. Employing dexamethasone peri‐procedurally may reduce tissue edema at the ablation site, although more clinical studies are needed to assess the efficacy of this drug.^17^

There were three limitations to our meta‐analysis. One limitation is that only observational studies were included. The number of patients was also small because of a lack of randomized controlled trial. No studies have compared radiofrequency septal ablation with more accepted therapies, such as ASA and myectomy. Although results from all published clinical studies were encouraging, results of long‐term outcomes including HCM‐related mortality, adverse arrhythmic events, permanent pacemaker dependency, and sudden cardiac death remained absent, and the follow‐up period was short in current published studies. Second, we did not calculate the percentage of persistent LVOT obstruction after the procedure and the incidence of reintervention because the definition of persistent LVOT obstruction varied among studies. Third, clinical heterogeneity might exist between the studies since they were performed in different countries, different populations (including children), and using different approaches (right ventricular and apex). Finally, as the data of this analysis were mainly descriptive, the conclusion must be drawn with caution.

The incidence of cardiomyopathy, especially HOCM, is low when compared with other cardiovascular diseases, such as hypertension, coronary artery disease, and atrial fibrillation.^1^ It is not surprising that the number of studies of radiofrequency septal ablation is small, as only staff in experienced centers conducts the procedure. However, data from the present clinical studies showed that radiofrequency septal ablation might become a promising invasive treatment for patients with HOCM who are still symptomatic despite receiving maximally tolerated drug therapy. Therefore, a large‐scale randomized controlled trial comparing radiofrequency septal ablation with ASA or myectomy is needed to further assess the efficacy and safety of radiofrequency ablation for patients with HOCM.

## 5 CONCLUSIONS

Radiofrequency septal ablation allows significant reduction in the LOVT gradient and symptomatic improvement of patients with HOCM. Although further study is required, this novel technique is safe and effective, which makes it a promising alternative approach in the treatment of patients who are unsuitable for undergoing myectomy or ASA.

## CONFLICT OF INTEREST

The authors declare no potential conflict of interests.

## Supporting information


**Appendix** **S1**: Supporting Information
