# Supplementary material for: Efficacy and safety of radiofrequency ablation for hypertrophic obstructive cardiomyopathy: A systematic review and meta‐analysis
Source: Clin Cardiol. 2020 Feb 7;43(5):450–8. doi: 10.1002/clc.23341 (PMC7244291; doi:10.1002/clc.23341)
Supplement: Supplementary file 1 — Appendix S1: Supporting Information [file CLC-43-450-s001.docx]

**Supplementary material**

The complete search terms was: Cardiomyopathy, Hypertrophic [Mesh] OR Cardiomyopathies, Hypertrophic OR Hypertrophic Cardiomyopathies OR Cardiomyopathy, Hypertrophic Obstructive OR Cardiomyopathies, Hypertrophic Obstructive OR Hypertrophic obstructive cardiomyopathy OR Hypertrophic obstructive cardiomyopathies OR Obstructive Cardiomyopathies, Hypertrophic OR Obstructive Cardiomyopathy, Hypertrophic OR Septal hypertrophy OR Left Ventricular Outflow Obstruction OR HCM OR HOCM AND Radiofrequency Ablation [Mesh] OR Ablation, Radiofrequency OR Radio Frequency Ablation OR Ablation, Radio Frequency OR Radio-Frequency Ablation OR Ablation, Radio-Frequency OR Endocardial radiofrequency ablation OR Radiofrequency catheter ablation OR Catheter ablation OR Radiofrequency.
